# Differential Effects of Hypothermia and SZR72 on Cerebral Kynurenine and Kynurenic Acid in a Piglet Model of Hypoxic–Ischemic Encephalopathy

**DOI:** 10.3390/ijms241914522

**Published:** 2023-09-25

**Authors:** Ferenc Domoki, Valéria Tóth-Szűki, Viktória Kovács, Gábor Remzső, Tímea Körmöczi, László Vécsei, Róbert Berkecz

**Affiliations:** 1 Department of Physiology, Albert Szent-Györgyi Medical School, University of Szeged, 6720 Szeged, Hungary; toth-szuki.valeria@med.u-szeged.hu (V.T.-S.); kovacs.viktoria.1@med.u-szeged.hu (V.K.); remzso.gabor@med.u-szeged.hu (G.R.); 2 Institute of Pharmaceutical Analysis, Interdisciplinary Excellence Center, University of Szeged, 6720 Szeged, Hungary; kormoczi.timea@szte.hu (T.K.); berkecz.robert@szte.hu (R.B.); 3 ELKH-SZTE-Neuroscience Research Group, Hungarian Academy of Sciences, University of Szeged, 6720 Szeged, Hungary; 4 Department of Neurology, Interdisciplinary Excellence Center, Albert Szent-Györgyi Medical School, University of Szeged, 6720 Szeged, Hungary

**Keywords:** hypoxia, neonate, tryptophan metabolism, kynurenine pathway, newborn pig

## Abstract

Kynurenic acid (KYNA), an endogenous neuroprotectant with antiexcitotoxic, antioxidant, and anti-inflammatory effects, is synthesized through the tryptophan-kynurenine (KYN) pathway. We investigated whether brain KYN or KYNA levels were affected by asphyxia in a translational piglet model of hypoxic–ischemic encephalopathy (HIE). We also studied brain levels of the putative blood–brain barrier (BBB) permeable neuroprotective KYNA analogue SZR72, and whether SZR72 or therapeutic hypothermia (TH) modified KYN or KYNA levels. KYN, KYNA, and SZR72 levels were determined using ultra-high-performance liquid chromatography coupled with tandem mass spectrometry in five brain regions 24 h after 20 min of asphyxia in vehicle-, SZR72- and TH-treated newborn piglets (n = 6-6-6) and naive controls (n = 4). Endogenous brain KYN levels (median range 311.2–965.6 pmol/g) exceeded KYNA concentrations (4.5–6.0 pmol/g) ~100-fold. Asphyxia significantly increased cerebral KYN and KYNA levels in all regions (1512.0–3273.9 and 16.9–21.2 pmol/g, respectively), increasing the KYN/Tryptophan-, but retaining the KYNA/KYN ratio. SZR72 treatment resulted in very high cerebral SZR72 levels (13.2–33.2 nmol/g); however, KYN and KYNA levels remained similar to those of the vehicle-treated animals. However, TH virtually ameliorated asphyxia-induced elevations in brain KYN and KYNA levels. The present study reports for the first time that the KYN pathway is altered during HIE development in the piglet. SZR72 readily crosses the BBB in piglets but fails to affect cerebral KYNA levels. Beneficial effects of TH may include restoration of the tryptophan metabolism to pre-asphyxia levels.

## 1. Introduction

Hypoxic–ischemic encephalopathy (HIE) is a major cause of neonatal mortality and long-term disabilities like epilepsy, neurocognitive impairments, and cerebral palsy [1,2]. HIE is caused by hypoxic/ischemic insults in the perinatal period such as intrapartum asphyxia in term neonates. The pathophysiology of brain injury during HIE development is a multistep process: during the primary hypoxic insult, the failed respiratory gas exchange and hemodynamic failure trigger cellular energy failure with intracellular Ca^++^ overload, excitoxicity, and severe combined acidosis. Reventilation/reoxygenation restores aerobic energy production but also triggers further oxidative stress [3]. These primary pathophysiological processes set the stage for the onset of delayed injury marked by secondary energy failure, seizures, and neuroinflammation [4,5]. Therapeutic hypothermia (TH) provides significant, albeit incomplete neuroprotection, reducing both mortality and the incidence of long-term disabilities [6]. Furthermore, TH treatment requires high-resource clinical settings often not available to medical staff treating HIE patients. Thus, adjunct therapies supplementing the neuroprotection afforded by TH, or alternative neuroprotective treatments that can be employed in low-resource settings, are required, necessitating further preclinical research. The newborn (postnatal day 1; P1) pig emerged as an excellent translational preclinical HIE model as its brain structure, brain developmental stage, cerebral blood flow, and energy metabolism closely approximates the human term neonate [7,8,9].

Tryptophan (TRP) is an essential amino acid that is metabolized in mammals either through the serotonin or the kynurenine (KYN) pathways. The serotonin pathway yields important neurotransmitters, and also the hormone melatonin. However, products of the KYN pathway are responsible for more than 90% of tryptophan metabolism [10], and its products, namely, kynurenic acid (KYNA) and quinolinic acid can also modulate neuronal activity, and the latter is also precursor to the important NAD^+^ coenzyme [11]. KYNA was found to be neuroprotective in a number of preclinical models including P6 rat HIE models. SZR72 (2-(2-*N*, *N*-dimethylaminoethylamine-1-carbonyl)-1*H*-quinolin-4-one hydrochloride) is a synthetic KYNA analogue developed to enhance KYNA’s poor blood–brain barrier penetration (BBB) [12]. SZR72 was indeed found to have a beneficial effect in rat models of a number of brain pathologies including global cerebral ischemia [13]. However, we have recently reported that SZR72 treatment initiated after asphyxia did not preserve neurons in the subacute (24 h) phase of HIE development in our piglet model, although TH indeed provided marked, albeit partial neuroprotection [14].

The activity of the KYN pathway can be altered by hypoxic/ischemic stress. Cardiac arrest resulted in early elevation of blood plasma KYNA levels in adult rats, pigs, and humans, and in pigs, the KYN pathway activation correlated with brain histopathological findings [15]. Blood plasma metabolomic analyses in piglets exposed to hypoxia and resuscitation, and also human neonates exposed to perinatal asphyxia, also indicated the involvement of the KYN pathway in the metabolic response to hypoxic/ischemic stress [16,17]. However, cerebral levels of KYNA or quinolinic acid were found to be unaltered in the early reperfusion period after cerebral ischemia in piglets [18].

The main objectives of the present study were to address the question of whether SZR72 passed the BBB in our piglet HIE model and whether asphyxia in combination with SZR72 or TH could significantly affect the KYN and/or KYNA levels 24 h after asphyxia. To address these questions, we used brain samples collected from our previous study [14], supplemented by samples obtained from controls for comparison. We provide evidence for sufficient BBB transport of SZR72 into the piglet brain as well as novel data demonstrating how asphyxia alters cerebral regional KYN and KYNA levels that are not modulated by SZR72 but by TH in the piglet.

## 2. Results

Physiological data such as the core body temperature, mean arterial blood pressure, heart rate, arterial blood gas, and blood sugar and lactate values for the vehicle-treated (VEH), TH-treated, and SZR72-treated groups have been published in detail previously [14]. Briefly, the values were within the physiological limits at baseline and throughout the 24 h observation period and did not differ among the three experimental groups, with the exception of body temperature and heart rate that were significantly lower in the TH group after asphyxia. Moreover, asphyxia also triggered similar cardiorespiratory and blood chemistry changes in all groups, exhibiting matching values considered clinically significant compared with birth asphyxia in human neonates.

### 2.1. SZR72 Levels

**Figure 1:**
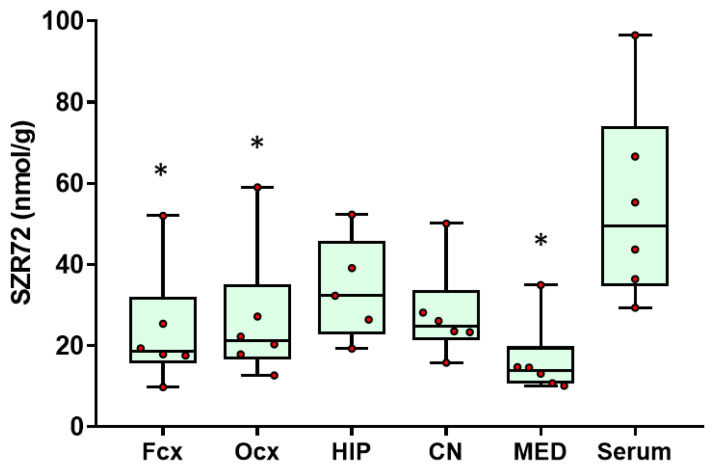
Brain and serum SZR72 levels. SZR72 treatment resulted in similar SZR72 levels in all examined brain regions (Fcx—frontal cortex, Ocx—occipital cortex, HIP—hippocampus, CN—caudate nucleus, MED—medulla oblongata), and there were no significant differences among the assessed brain regions. The data from the individual brain regions were also compared with serum levels obtained at 12–24 h after beginning of SZR72 administration, and the data indicating good blood–brain barrier penetration, as SZR72 concentrations in the hippocampus and the caudate were not significantly different from the serum values. Lines, boxes, and whiskers represent the median, the interquartile range, and the 10th–90th percentiles, respectively. * *p* < 0.05 significantly different from the serum values.

SZR72 was found in high concentrations (median range 13.87–32.31 nmol/g) in all brain regions studied from the SZR72 group, and the values were similar in all brain regions studied (Figure 1). Brain levels were merely 35–70% lower than serum concentrations (median 49.51 nmol/g), suggesting good BBB penetration. 

### 2.2. KYN Levels

**Figure 2:**
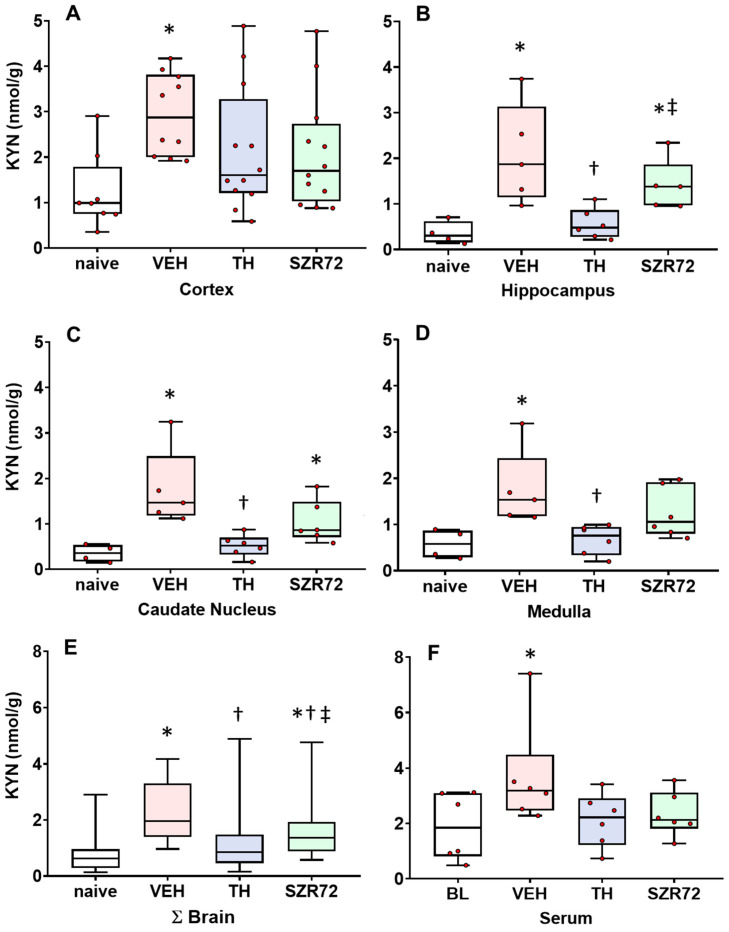
Asphyxia-induced changes in brain KYN levels. Panel (**A**) shows cerebrocortical kynurenine (KYN) levels in the naive controls, as well as in the vehicle (VEH), hypothermia-treated (TH), and SZR72-treated (SZR72) groups subjected to asphyxia. Panels (**B**–**E**) show the values obtained from the hippocampus, the caudate nucleus, the medulla, and the combined data obtained from all assessed brain regions (ΣBrain), respectively. Panel (**F**) shows the combined serum data from the asphyxiated groups collected at 12–24 h after asphyxia, while baseline data (BL) were obtained from the VEH group collected before exposure to asphyxia. KYN levels increased significantly after asphyxia in all brain regions in the VEH group compared with controls. In contrast, KYN levels in the TH group were significantly reduced compared with the VEH group in the hippocampus, the caudate nucleus, and the medulla, while the trend was non-significant in the cerebral cortex. In fact, TH appeared to reduce KYN levels to control (naive) levels. SZR72 had a modest effect on asphyxia-induced KYN levels, but the trend was found significant only if all brain regions were considered together. Importantly, TH resulted in significantly lower KYN levels in the hippocampus and the caudate nucleus compared with SZR72 treatment. Lines, boxes, and whiskers represent the median, the interquartile range, and the 10th–90th percentiles, respectively. * *p* < 0.05, * significantly different from the naive, † from the VEH, ‡ from the TH group.

In the untreated control (naive) animals, brain KYN levels were typically in the 0.35–0.99 nmol/g concentration range with a tendency of higher values in the cerebrocortical areas (frontal and occipital cortices). However, serum KYN levels were higher (median 1.85 nmol/g). Asphyxia elicited significant KYN concentration increases in all brain regions in the VEH group, and in the blood (Figure 2), and the data were normalized for the respective values from the naïve control group to allow evaluation of proportional changes and are shown in Supplementary Figure S1. Compared with the increases observed in the VEH group, TH significantly attenuated asphyxia-induced KYN increases in the hippocampus, caudate nucleus, and the medulla. SZR72 had a similar effect, however, it was statistically significant only when data from all areas were combined. 

### 2.3. KYNA Levels

**Figure 3:**
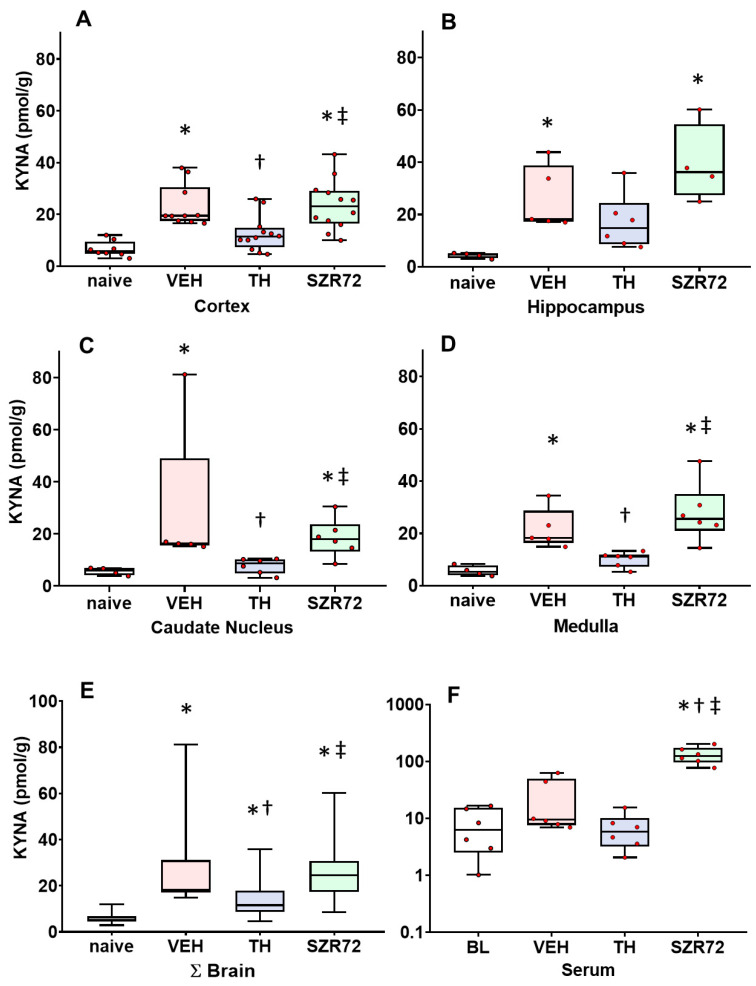
Asphyxia-induced changes in brain kynurenic acid (KYNA) levels. Panel (**A**) shows cerebrocortical KYNA levels in the naive controls, as well as in the vehicle (VEH), hypothermia-treated (TH), and SZR72-treated (SZR72) groups subjected to asphyxia. Panels (**B**–**E**) show the values obtained from the hippocampus, the caudate nucleus, the medulla, and the combined data obtained from all assessed brain regions (ΣBrain), respectively. Panel (**F**) shows the combined serum data from the asphyxiated groups collected at 12–24 h after asphyxia, while baseline data (BL) were obtained from the VEH group collected before exposure to asphyxia. KYNA levels increased significantly after asphyxia in all brain regions in the VEH group compared with controls. In contrast, KYN levels in the TH group were significantly reduced compared with the VEH group in the cortex and the medulla, while the trend was non-significant in the hippocampus and the caudate nucleus. SZR72 did not elevate brain KYNA levels, which were similar to the values of the VEH group in all brain regions. However, SZR72 greatly increased serum KYNA levels; please note the logarithmic scale on the Panel F graph. Lines, boxes, and whiskers represent the median, the interquartile range, and the 10th–90th percentiles, respectively. * *p* < 0.05, * significantly different from the naive, † from the VEH, ‡ from the TH group.

In the naive animals, brain KYNA levels were typically two orders of magnitudes lower than KYN concentrations, and this was similar among the assessed brain regions (the median range was 4.6–6.3 pmol/g) and the serum (6.29 pmol/g). However, KYNA levels were also elevated via asphyxia in all brain regions, but not in the blood in the VEH group (Figure 3). TH ameliorated asphyxia-induced KYNA increases in most brain regions, and the effect was statistically significant in the cerebral cortex and the medulla. TH did not affect serum KYNA levels. Interestingly, SZR72 treatment could not elevate asphyxia-induced brain KYNA levels further, although it did increase serum KYNA levels more than 10-fold (Figure 3); the data were normalized to the respective values from the naïve control group to allow evaluation of proportional changes and are shown in Supplementary Figure S2.

### 2.4. KYNA/KYN and KYN/TRP Ratios

**Figure 4:**
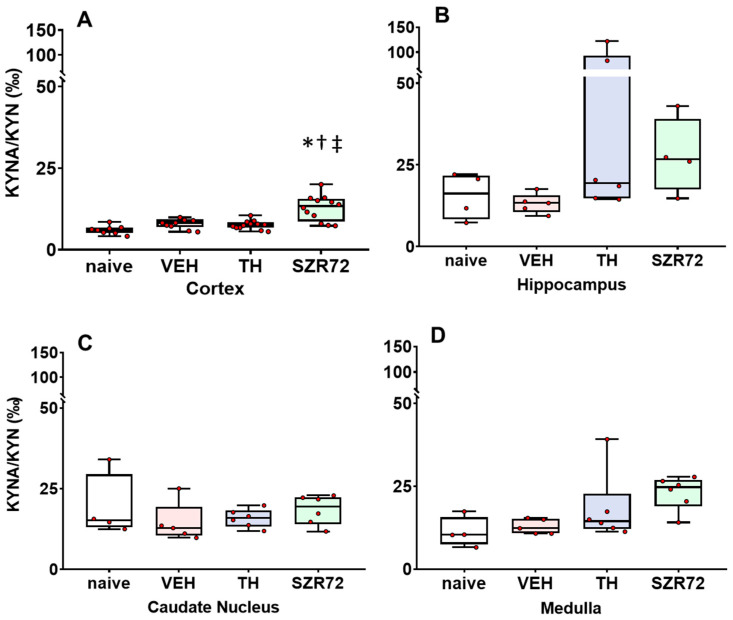
Asphyxia-induced changes in brain KYNA/KYN ratios. Panel (**A**) shows cerebrocortical KYNA/KYN ratios in the naive controls, as well as in the vehicle (VEH), hypothermia-treated (TH), and SZR72-treated (SZR72) groups subjected to asphyxia. Panels (**B**–**D**) show the values obtained from the hippocampus, the caudate nucleus, and the medulla, respectively. KYN/KYNA ratio increased moderately, but significantly only in the cerebral cortex of the SZR72-treated group compared with all other groups. Otherwise, KYNA/KYN ratios were not significantly different in any other brain regions among the experimental groups. Lines, boxes, and whiskers represent the median, the interquartile range, and the 10th–90th percentiles, respectively. * *p* < 0.05, * significantly different from the naive, † from the VEH, ‡ from the TH group.

**Figure 5:**
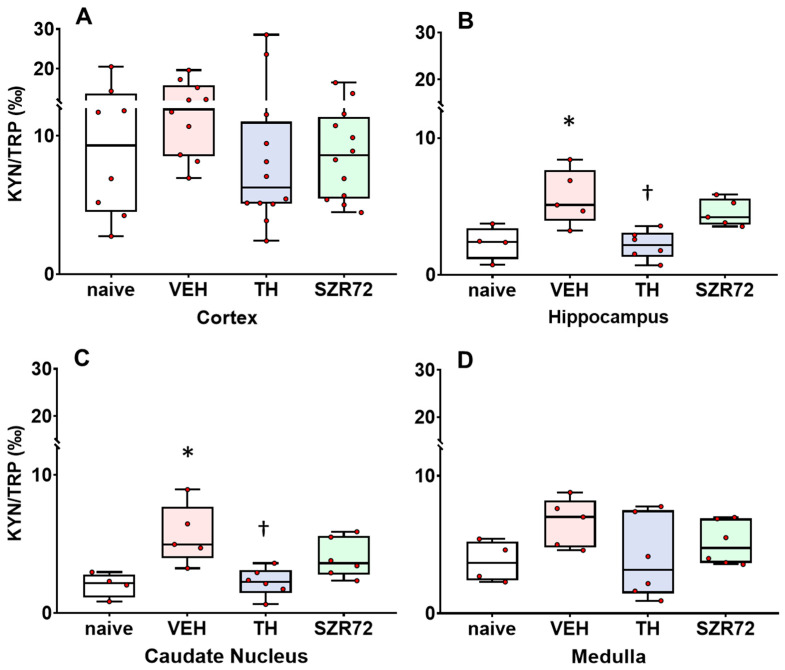
Asphyxia-induced changes in brain kynurenine/tryptophan (KYN/TRP) ratios. Panel (**A**) shows cerebrocortical KYN/TRP ratios in the naive controls, as well as in the vehicle (VEH), hypothermia-treated (TH), and SZR72-treated (SZR72) groups subjected to asphyxia. Panels (**B**–**D**) show the values obtained from the hippocampus, the caudate nucleus, and the medulla, respectively. KYN/TRP ratios increased significantly after asphyxia in the hippocampus and the caudate nucleus, but a similar tendency could be observed in the other brain regions as well. Moreover, in the hippocampus and the caudate nucleus, TH reduced the KYN/TRP ratio back to control levels, while SZR72 did not have such a pronounced effect. Lines, boxes, and whiskers represent the median, the interquartile range, and the 10th–90th percentiles, respectively. *p < 0.05, * significantly different from the naive, † from the VEH group.

In the assessed brain regions, KYNA/KYN ratios were similar between the different groups, and the KYNA/KYN ratio was moderately, but significantly increased in the SZR72 group only in the cerebral cortex (Figure 4). In contrast, KYN/TRP ratios were affected by asphyxia compared with the naïve controls and asphyxia in the VEH group significantly increased the KYN/TRP ratio in the hippocampus and the caudate nucleus, while the corresponding tendency in the cortex and the medulla was not significant (Figure 5). TH, but not SZR72 treatment antagonized this effect of asphyxia.

### 2.5. Spectral Edge Frequency (SEF)

**Figure 6:**
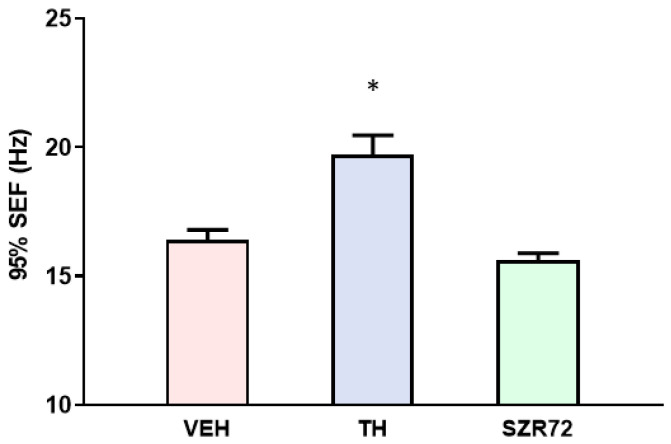
Graph showing 95% spectral edge frequency (SEF) values in the experimental groups subjected to asphyxia. 95% SEF represents the EEG frequency below which 95% of EEG power resides. TH treatment resulted in significantly higher 95% SEF compared with the VEH group, while SZR72 treatment had no such effect. Data are shown mean ± SEM, * *p* < 0.05, significantly different from the VEH group.

We reported previously that the total EEG power was enhanced by SZR72, but not by TH 12–24 h after asphyxia [14]. However, we now present the finding that this increased EEG power is dominated by low-frequency activity, expressed by calculating 95% SEF (Figure 6). Indeed, 95% SEF values of the SZR72 groups were significantly lower than those of the TH group and similar to those of the VEH group, in agreement with the previously reported lack of neuroprotection offered by SZR72 and the neuroprotection afforded by TH. 

## 3. Discussion

The present study significantly advances our knowledge on the applicability of synthetic KYNA analogues as neuroprotective agents in the treatment of HIE, using a translational preclinical HIE model, the newborn pig. In our model, HIE was elicited with asphyxia, which was in accordance with the clinical definition of severe birth asphyxia in humans and in piglets [19,20]. Using this model, we demonstrated previously that this level of asphyxia was able to elicit marked HIE, shown by significant neuronal damage in the most vulnerable regions such as the basal ganglia, the cerebral cortex, and the hippocampus [14]. This neuronal damage was partially prevented by TH, showing that the model was appropriate for detecting the neuroprotective efficacy of new therapies. However, SZR72 treatment initiated after asphyxia, using a dose that simulated an effective neuroprotective dose established in a rat cerebral ischemia model, failed to exhibit neuroprotection in our HIE model; however, the mechanism behind the lack of effect remained unclear [14].

SZR72 has been developed to circumvent the poor BBB penetration of exogenously administered KYNA in order to enhance bioavailability in the brain [12]. Despite numerous published studies on the beneficial effects of SZR72 in various preclinical disease models, brain SZR72 levels, or other indicators of in vivo BBB permeability after systemic administration of SZR72, remained unreported [13]. One of the most important findings in our present study is the demonstration of the very good in vivo BBB penetration of SZR72, in accordance with the implications and unpublished observations of previous studies using SZR72 [12]. Our present study does not allow us to speculate on the kinetics of SZR72 movement across the BBB, as brain samples were collected 24 h after the initiation of SZR72 treatment, however, data in the literature suggest fast BBB permeation: in a rat electrophysiological study, hippocampal CA1 field excitatory postsynaptic potentials and the induction of long-term potentiation were significantly altered already 2–3 min after *ip* administration of SZR72, but not KYNA [21]. In light of our present findings showing high brain SZR72 levels, we can imply that the faster recovery of EEG power, compared with the VEH and TH groups, that was reported previously [14] was likely due to the high brain SZR72 levels that developed within a few hours after administration. This likely exerted a stimulatory effect on neuronal activity similar to what was described previously in the case of KYNA [22].

Further, the present study also confirms our previous observations that the BBB remained intact in our HIE model. We previously demonstrated that a large pH gradient (0.9–1.0 pH unit) developed between the brain extracellular compartment and the blood during asphyxia, and that it was then gradually restored to normal values in the re-oxygenation period [23]. In the present study, the intact BBB function is suggested by the large KYNA concentration gradient that developed across the BBB in the SZR72-treated animals, as SZR72 elevated only blood, but not brain KYNA levels, and apparently, the elevated blood KYNA was not followed by an increase in brain KYNA levels; this suggests a KYNA-impermeable BBB. The cause of the increase in blood KYNA levels in the SZR72 group remains unexplored. Despite this increase in blood KYNA concentration, blood SZR72 levels still exceeded KYNA by ~500 times. As blood KYN levels were unaffected by the SZR72 treatment, this increase in blood KYNA levels is unlikely to represent an increase in endogenous KYNA production through the KYN pathway, but rather a peripheral conversion of a small percentage of SZR72 into KYNA. Hepatic amidases might be responsible, as they are known to hydrolyze amide bonds in procainamide with a similar structure to SZR72 [24]. Alternatively, the high plasma concentration of SZR72 could affect renal KYNA excretion. Renal handling of SZR72 is unexplored, but KYNA has been reported to be a subject of renal tubular secretion via organic anion transporters [25], so high SZR72 levels could also compete with KYNA at these transporters that reduce KYNA excretion. 

In light of the findings of a large SZR72 concentration developing in the piglet brain after *iv* SZR72 administration, the conspicuous lack of neuroprotection by SZR72 in our HIE model remains intriguing. Perhaps not SZR72, *per se*, but SZR72-derived KYNA produced locally in the brain could cause neuroprotection, and differential age/species-dependent metabolic routes of SZR72 are responsible for the strikingly different outcomes between adult rat and newborn piglet studies [13,14]. Alternatively, the SZR72- or KYNA-induced neuroprotection in rat HIE models is thought to be chiefly mediated by the antioxidant [26] and antiexcitotoxic effects of KYNA [27], and NMDA-receptors are indeed thought to play an important role in neuronal injury in the piglet HIE model as well [28]. However, the antiexcitotoxic effect of KYNA/SZR72 may not be sufficient to reduce neuronal cell death in our HIE models, as recent studies suggest that alternative cell death pathways can be activated in neonatal hypoxic/ischemic injuries in case of antiexcitotoxic treatments, if the upstream triggers of neuronal injury are strong enough [29]. Finally, it is possible that, compared with pretreatments, the SZR72 treatment initiated after the hypoxic/ischemic insult also initiates detrimental effects that neutralize neuroprotection. In the present study, the nearly identical 95% SEF values in the VEH and SZR72 groups suggest this outcome is a possibility [30]. This possibility is strongly supported by previous studies that aimed to increase cerebral KYNA levels through administration of its BBB-permeable precursor, KYN-sulphate (KYNS). Although KYNS pretreatment indeed did afford neuroprotection in a rat global cerebral ischemia model [31], postischemic administration of KYNS in a rat transient middle cerebral artery occlusion model exacerbated the neuronal damage instead [32]. Despite the lack of effect under the present conditions, SZR72 and perhaps newer analogues will be more useful neuroprotective drugs in HIE models featuring common clinical complications like chorioamnionitis and/or neonatal sepsis when TH is not efficient [33]. SZR72 was found to ameliorate brain damage associated with sepsis in adult rats [34], and newer KYNA derivatives can elicit profound anti-inflammatory actions through effects on microglia [35]. 

The other major finding of the present study is that it provides novel evidence that brain KYN and KYNA levels are increased after hypoxic/ischemic stress in a gyrencephalic large animal piglet model in the subacute phase of HIE development. Previous data from piglets suggested that KYNA levels are unchanged in the acute (0.5–2 h) reperfusion period after global cerebral ischemia [18]. In contrast, asphyxia induced major increases in brain KYNA levels in the acute (~10 min) re-oxygenation period in neonatal rats, and furthermore, the KYNA level increases were dependent on the duration of the asphyxia [36]. Similar confounding reports stated unchanged hippocampal KYNA levels after ischemia in adult gerbils [37], but immunohistochemistry in rats identified elevated numbers of KYNA-expressing astrocytes in the subacute period after focal cerebral ischemia as well [38]. In contrast to the equivocal findings concerning brain-level KYNA responses to hypoxic/ischemic insults, studies on blood KYNA responses are more straightforward. The rapid activation of the KYN pathway with the elevation of its metabolites has also been shown after cardiac arrest in adult rats, pigs, and humans [15]. Even more importantly, a clinical study found that blood KYN and KYNA levels obtained from human HIE patients at 6 or 24 h after birth asphyxia were higher than samples obtained at later time points, which also indicates the early activation and the slow restoration of the KYN pathway activity in human neonates exposed to hypoxic/ischemic stress [39]. Our present findings of essentially unchanged KYNA/KYN, but significantly increased KYN/TRP ratios in the most asphyxia-vulnerable regions of the piglet brain suggest that asphyxia and/or reoxygenation primarily triggers enhanced TRP metabolism into KYN, while the observed increase in brain KYNA levels may be secondary to the increased substrate availability to the astrocytes that are mainly responsible for KYNA production in the central nervous system [40]. Our findings are in agreement with previous data obtained in a mouse stroke model using middle cerebral artery occlusion [41]. In that study, stroke-induced brain KYN increases have been demonstrated to be dependent on the activity of TRP 2,3-dyoxgenase, the KYN-synthesizing enzyme known to be expressed in the liver and in neurons [42,43]. In that model, TDO was shown to be induced by cerebral ischemia, and pharmacological inhibition of TDO was found to be neuroprotective [41]. Exogenous KYN reversed the neuroprotective effect, and the neurotoxic effect of KYN was shown to be mediated by the activation of the aryl-hydrocarbon receptor (AhR), as KYN is an endogenous ligand of this transcription factor involved in tumor cell progression and in promoting neuronal cell death [44,45]. The increases in brain KYN levels observed in our HIE model could conceivably trigger similar neurotoxicity through AhR activation, although this possibility is yet unexplored in the piglet HIE model; however, brain expression of AhR in the pig has been established [46]. Interestingly, the neuroprotective TH treatment attenuated the asphyxia-induced changes both in KYN levels and KYN/TRP ratios, which may constitute one of the neuroprotective mechanisms of TH. However, it is also conceivable that KYN level changes were attenuated by TH through exerting neuroprotective effects unrelated to the TRP-KYN-KYNA pathway, and that the attenuation simply reflects the smaller injury caused by asphyxia in the TH group. KYNA could affect neuronal activity and survival in addition to the already mentioned glutamatergic receptors. α7 nicotinic acetyl-choline receptors were shown to be inhibited by KYNA [47], although this KYNA mechanism of action has recently been debated [48]. Furthermore, KYNA is known to activate the G-protein-coupled receptor 35 (GPR35) which has been demonstrated to be expressed in hippocampal neurons [49] regulating neuronal activity; however, GPR35 expressed widely in immune cells can be an important regulator coupling the KYN pathway to neuroimmune communication [50]. Our findings suggest that the observed increases in endogenous brain KYNA levels are unable to elicit significant neuroprotective actions, and SZR72—albeit present in pharmacological concentrations—does not activate these supposedly neuroprotective targets in sufficient amounts to exert observable neuroprotection when compared with TH in our HIE model. 

This study has a number of important limitations. The pharmacological actions of SZR72 itself on KYN or KYNA targets such as AhR, α7 nicotinic acetyl-choline receptor, and GPR35 are unexplored. The enzyme levels or activities governing the KYN pathway remain unexplored in this experimental model. Furthermore, the causative vs. coincidental nature of the effects of TH on changes in KYN or KYNA levels cannot be unequivocally determined. 

## 4. Materials and Methods

### 4.1. Animals

Newborn (P1) male Landrace piglets (n = 22, weighing between: 1.5–2.5 kg) were obtained from a local company (Pigmark Ltd., Co., Szeged, Hungary) and delivered to the laboratory on the morning of the experiments. The experimental procedures and protocols, including obtaining and analyzing KYN, KYNA, and SZR72 levels in serum samples for the VEH, TH, and SZR72 groups (n = 6-6-6) have been previously described in detail [14]. Briefly, the anesthesia was induced with sodium thiopental (*ip*, 45 mg/kg; Sandoz, Kundl, Austria) and maintained throughout the experiment with morphine and midazolam infusion (*iv*, 10 and 250 μg/kg/h, Teva, Petach Tikva, Israel and Torrex Pharma, Vienna, Austria, respectively). The animals were intubated through a tracheotomy, mechanically ventilated, and the right femoral vein and carotid artery were catheterized for the administration of fluids and to obtain arterial blood samples, respectively. Then, the instrumented animals were exposed to 20 min of asphyxia via ventilation with a gas mixture containing 6% O_2_ and 20% CO_2_. Asphyxia was followed by vehicle treatment (VEH), cooling the core temperature to 33.5 °C (TH), or SZR72 treatment (170 mg/kg *iv* bolus followed by 150 mg/kg/12 h *iv* infusion), and observation was carried out for 24 h while recording physiological parameters, blood gases, and the EEG. To obtain baseline brain KYN and KYNA levels for the present study, an additional naive (untreated control) group (n = 4) was utilized. These animals were anesthetized with sodium thiopental and mechanically ventilated similarly to the three groups exposed to asphyxia, but they were immediately (not at the end of the 24 h observation period after asphyxia) processed for brain sampling. In this procedure, first, both carotid arteries of the anesthetized animals were catheterized in the distal direction. Then, the animals of all four groups were euthanized with pentobarbital sodium (300 mg, Release^®^; Wirtschaftsgenossenschaft deutscher Tierärzte eG, Garbsen, Germany), and the brains were perfused with cold (4 °C) physiological saline, similar in all groups. The brains were gently removed from the skull, and brain samples from the frontal and the occipital cortices, the hippocampus, the caudate nucleus, and the medulla were collected and stored as described in Section 4.3.

### 4.2. Materials 

LC-MS grade water (H_2_O), methanol (MeOH), and formic acid (FA) were from VWR (Radnor, PA, USA). Kynurenic acid-d5 (KYNA-D5) was purchased from Toronto Research Chemicals (Toronto, ON, Canada), while TRP, KYNA, and KYN from Sigma-Aldrich (St. Louis, MO, USA). The SZR72 and SZR73 KYNA analogues were provided by the Institute of Pharmaceutical Chemistry of the University of Szeged.

### 4.3. Sample Preparation of Brain Regions for Quantitative Analysis

The wet brain samples were weighed and snap-frozen in liquid nitrogen and stored at –80 °C until homogenization. Two sample preparation procedures were developed and applied; one was for determining KYN and KYNA levels, while the other was used for SZR72 because of significantly different concentrations.

First, 100 mg of the brain samples was placed into 1.7-mL microcentrifuge tubes and an appropriate volume of homogenizing solution (5 mM EDTA, 2.5 mM ascorbic acid, pH 5.5) was added to obtain 7.67 mg/mL concentration of each brain homogenate. Then, the samples were individually sonicated with an ultrasonic homogenizer (BioLogics Model 150VT, BioLogics Inc., Manassas, VA, USA) for 4 min using a 20% power setting on the micro-tip probe and a 70% pulse on ice.

For the preparation and dilution of the standard stock solution, MeOH/2.5 mM aqueous ascorbic acid (50/50, *v*/*v*) solution (working solution) was used. A total of 1 mg of the standards was dissolved in 1 mL of the following solutions: KYN in working solution/FA solution (99/1, *v*/*v*), KYNA and KYNA-D5 in working solution/4% ammonia solution (90/10, *v*/*v*/*v*), and SZR72 and SZR73 in working solution. The sample preparation procedure of endogenous KYN and KYNA was as follows: First, 120 µL brain homogenate was spiked with 10 µL internal standard (IS-1) solution containing 50 nM KYNA-D5 in working solution, and 10 µL working solution, instead of calibration mixture was produced, then vortexed. For protein precipitation, 5 µL TFA was added to the spiked homogenate. The homogenate was vortex mixed for 15 s, and then it was ultrasonicated for 2 min at 4 °C, followed by sample centrifugation at 15,000 rpm for 15 min at 4 °C (Hettich 320R, Tuttlingen, Germany). Then, 120 µL of the upper layer was transferred to a 250 µL conical insert for analytical measurement. For calibration samples, 120 µL pulled control brain homogenate was spiked with 10 µL IS-1, then 10 µL given calibration mixture containing KYN and KYNA in MeOH/2.5 mM aqueous ascorbic acid solution (50/50, *v*/*v*) was added, and then, 5 µL TFA. Then, sample preparation was followed by the sample preparation protocol described above. The calibration points were the following: 0, 597.7, 1087, 2174, 3262, 4349, and 5436 pmol/g brain for KYN, and 0, 10.87, 21.74, 32.62, 43.49, 65.23, and 86.97 pmol/g brain for KYNA.

For the analysis of SZR72, the sample preparation procedure detailed above was applied with some modifications. Briefly, 10 µL (IS-2) solution containing SZR73 (1.67 µM) in working solution, 10 µL working solution, or calibration solution of SZR72 for the calibration sample as well as 190 µL saline were added to 10 µL homogenate in a microcentrifuge tube. Then, 10 µL of TFA was used for protein precipitation. Additional steps were the same as detailed above, except collecting 200 µL of the upper phase for analysis. For calibration of SZR-72, 0, 6.523, 13.05, 32.62, 65.23, 97.85, and 130.5 nmol/g brain calibration points were used.

### 4.4. Targeted Ultra-High-Performance Liquid Chromatography Coupled with High-Resolution Tandem Mass Spectrometry (UHPLC–MS/HRMS) Parameters for Quantitation of KYN, TRP, KYNA, and SZR72

UHPLC-MS/HRMS measurements were performed on Waters Acquity I-Class UPLC (Milford, MA, USA), connected to Thermo Scientific Q Exactive Plus Hybrid Quadrupole-Orbitrap™ (Waltham, MA, USA) mass spectrometer. The UPLC system was controlled by the MassLynx software V4.1 SCN805 (Milford, MA, USA). Data were acquired and evaluated with Xcalibur 4.2. software (Thermo Fisher Scientific, Waltham, MA, USA).

UHPLC separations were carried out on Kinetex biphenyl analytical column [100 × 2.1 mm, 2.6 μm, from Phenomenex (Torrance, CA, USA)]. The UHPLC mobile phase *A* consisted of 1% formic acid aqueous solution and mobile phase *B* was composed of MeOH with 1% formic acid. The gradient program was the following: 0–1 min 3% *B*, 4.5–6 min 100% *B*, and 6.1–9 min 3% *B*. The flow rate gradient program was 0–4.5 min 0.3 mL/min, 4.51–6 min 0.6 mL/min, 6.1–7.8 min 0.5 mL/min, and 7.81–8 min 0.3 mL/min. Column temperature was maintained at 50 °C, and the autosampler temperature and injection volume were set to 15 °C, and 7 µL, respectively.

For MS detection of targeted compounds, positive heated-electrospray ionization (H-ESI) was used with the following parameters: capillary temperature 270 °C, S-Lens RF level 50, spray voltage 4.5 kV, sheath gas flow 50, spare gas flow 2.5, and auxiliary gas flow 12.50 in arbitrary unit. In scheduled parallel reaction monitoring (PRM) with a resolution of 17,500 (FWHM), the AGC setting was defined as 3 × 10^6^ charges and the maximum IT was set to 50 ms. The width of the isolation window of the precursor ion was 1.0 Da. The optimized collision energies (CE) with the main UHPLC-MS/HRMS parameter are shown in Table 1 [51,52]. The effluent was introduced into the H-ESI source via a 2-position, 6-port divert valve only in the time range of 2–4.5 min. In the rest of the total run time, the H-ESI source was rinsed with acetonitrile/water solution (90/10, *v*/*v*) at a flow rate of 0.2 mL/min using Waters Acquity I-Class pump (Milford, MA, USA).

The standard addition in combination with the external calibration method was used for the quantitative analysis of the endogenous KYN and KYNA, while external calibration was applied for the quantitation of exogenous SZR72. In both calibration approaches, the peak areas of targeted compounds were normalized to the peak area of the given internal standard such as KYN, TRP, and KYNA to KYNA-D5, and SZR72 to SZR73. The TRP was semi-quantitatively analyzed, and the KYN/TRP values were determined by the normalized peak area ratios of KYN and TRP.

### 4.5. Spectral Edge Frequency (SEF) Analysis 

The methodology for EEG data collection and determination of power spectrum density (PSD) was detailed previously [14]. Briefly, the PSD data were determined using the Welch method in MATLAB environment with custom-written scripts, built-in functions, and the EEGlab toolbox [53]. From these data, the 95% SEF at 24 h after asphyxia was calculated. The 95% SEF can be defined as the threshold EEG frequency below 95% of the EEG spectral power resides. 

### 4.6. Statistics

Statistics were conducted using IBM SPSS 22.0 (IBM Corp., Armon, NY, USA), and GraphPad Prism 8.01 (Dotmatics, Boston, MA, USA). Non-normally distributed KYN/KYNA/SZR72 concentration values were analyzed with the Kruskal–Wallis analysis of ranks followed by the uncorrected Dunn’s *post hoc* test for all-pairwise comparisons. The 95% SEF data were analyzed with one-way ANOVA followed by the Tukey *post hoc* test. *p* values < 0.05 were considered statistically significant. 

## 5. Conclusions

In a translational preclinical model of hypoxic–ischemic encephalopathy, we provide novel evidence on the excellent bioavailability of the KYNA analogue SZR72 in all assessed brain areas, proving SZR72′s BBB permeability. SZR72 increased blood, but not brain KYNA concentrations, and thus, SZR72 is likely not metabolized in the brain into KYNA, and the lack of neuroprotection provided by SZR72 can likely be attributed to the lack of KYNA-like neuroprotective actions and a direct neuronal stimulatory effect. This study also provides novel evidence on the activation of the KYN pathway in the central nervous system during the early phase of HIE development in this experimental model, and the ability of TH to reduce this response can be associated with its neuroprotective effect. 

## Figures and Tables

**Table 1:** Main UHPLC-MS/HRMS parameters.

Targeted Compound	Retention Time (min)	Precursor Ion (*m*/*z*)	Main Fragment Ion (*m*/*z*)	Collision Energy (eV)
**KYN**	2.75	209.09207	192.06552	16
**TRP**	2.86	205.09715	188.07061	10
**KYNA**	3.41	190.04987	162.05496	20
**KYNA-D5** (IS *-1)	3.41	195.08125	167.05615	20
**SZR72**	2.98	260.13935	162.05496	38
**SZR73** (IS *-2)	3.07	274.15500	162.05496	38

* IS—internal standard.

## Data Availability

The data presented in this study are openly available in [Domoki, Ferenc; Tóth-Szűki, Valéria; Kovács, Viktória; Remzső, Gábor; Körmöczi, Tímea; Vécsei, László; Berkecz, Róbert (2023), “Cerebral kynurenic acid levels are increased by asphyxia but not by its putative neuroprotectant analogue SZR72 in a piglet model of hypoxic–ischemic encephalopathy”, Mendeley Data, V1, at [https://doi.org/10.17632/sx3fkmfrwn.1 (accessed on 21 September 2023)].

## Supplementary Materials

The supporting information can be downloaded at: https://www.mdpi.com/article/10.3390/ijms241914522/s1.
