# Supplementary material for: Differential Effects of Hypothermia and SZR72 on Cerebral Kynurenine and Kynurenic Acid in a Piglet Model of Hypoxic–Ischemic Encephalopathy
Source: Int J Mol Sci. 2023 Sep 25;24(19):14522. doi: 10.3390/ijms241914522 (PMC10572886; doi:10.3390/ijms241914522)
Supplement: Supplementary file 1 [file ijms-24-14522-s001.zip › ijms-2574781-supplementary.pdf]

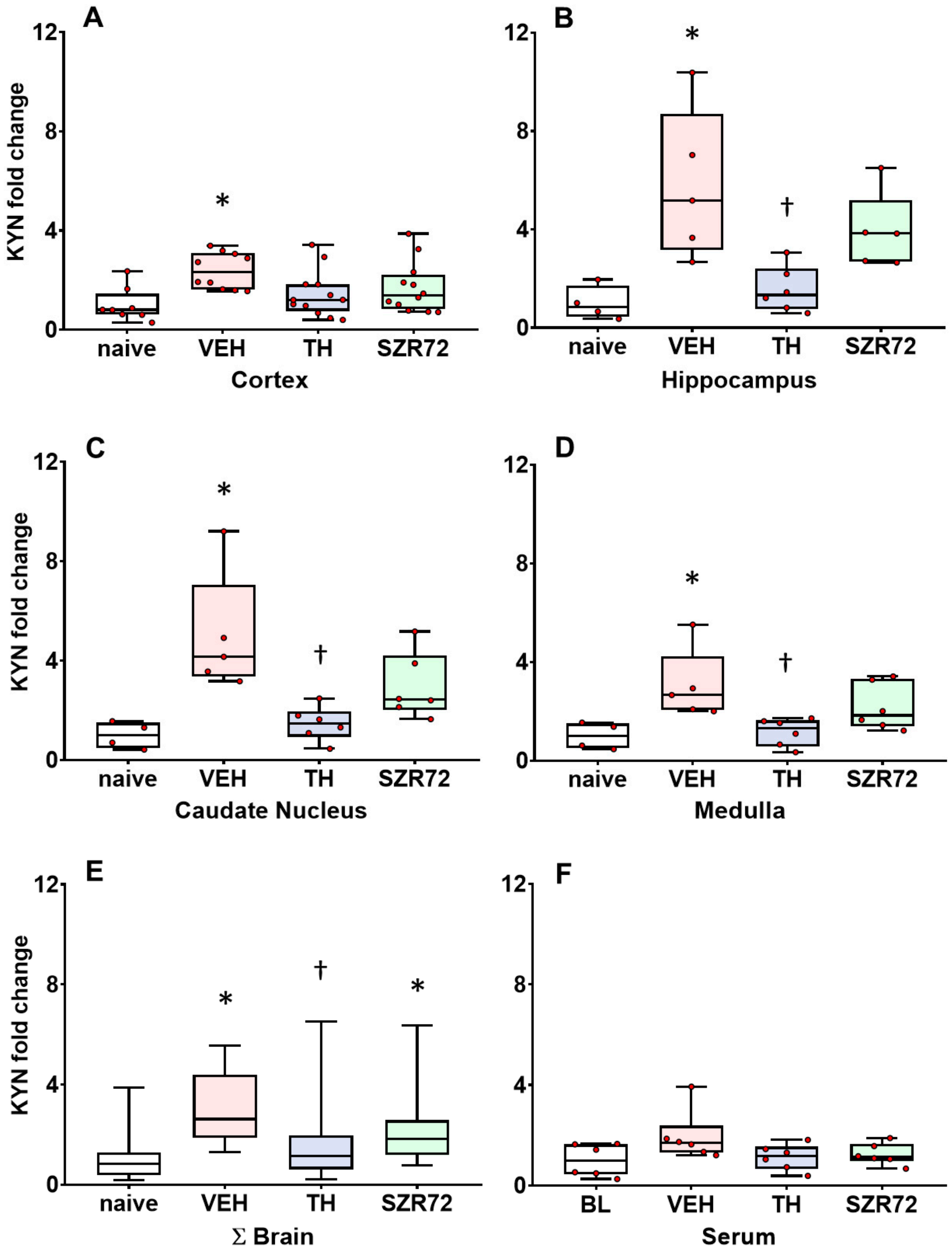

Supplementary Figure S1. Asphyxia-induced changes in brain KYN levels normalized for baseline levels determined in naïve controls. Panel A shows cerebrocortical KYN levels in the naive controls, as well as in the vehicle (VEH), hypothermia-treated (TH), and SZR72-treated (SZR72) groups subjected to asphyxia. Panels B-C-D and E show the values obtained from the hippocampus, the caudate nucleus, the medulla, and the combined data obtained from all assessed brain regions (ΣBrain), respectively. Panel F shows the combined serum data from the asphyxiated groups collected at 12-24h after asphyxia, while baseline data (BL) were obtained from the VEH group collected before exposure to asphyxia. KYN levels increased significantly after asphyxia in all brain regions in the VEH group compared with controls. In contrast, KYN levels in the TH group were significantly reduced compared with the VEH group in the hippocampus, the caudate nucleus, and the medulla, while the trend was non-significant in the cerebral cortex. In fact, TH appeared to reduce KYN levels to control (naive) levels. Lines, boxes, and whiskers represent the median, the interquartile range, and the 10th–90th percentiles, respectively. *$p < 0.05$, * significantly different from the naive, † from the VEH, ‡ from the TH group.

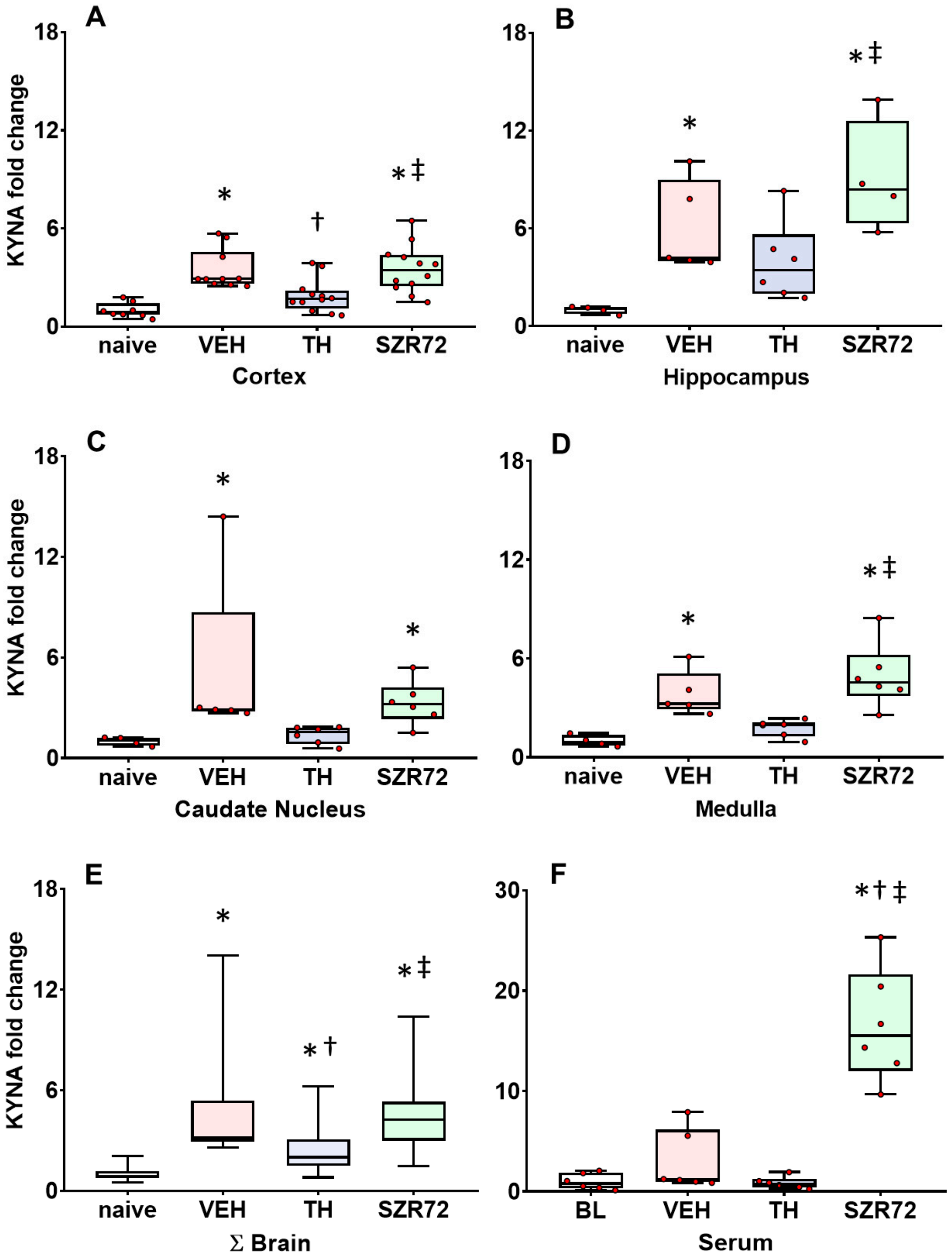

Supplementary Figure S2. Asphyxia-induced changes in brain KYNA levels normalized for baseline levels determined in naïve controls. Panel A shows cerebrocortical KYNA levels in the naive controls, as well as in the vehicle (VEH), hypothermia-treated (TH), and SZR72-treated (SZR72) groups subjected to asphyxia. Panels B-C-D and E show the values obtained from the hippocampus, the caudate nucleus, the medulla, and the combined data obtained from all assessed brain regions (ΣBrain), respectively. Panel F shows the combined serum data from the asphyxiated groups collected at 12-24h after asphyxia, while baseline data (BL) were obtained from the VEH group collected before exposure to asphyxia. KYNA levels increased significantly after asphyxia in all brain regions in the VEH group compared with controls. In contrast, KYNA levels in the TH group were significantly reduced compared with the VEH group in the cortex, while the corresponding trend was non-significant in the other brain regions. SZR72 did not elevate brain KYNA levels, which were similar to the values of the VEH group in all brain regions. However, SZR72 greatly increased serum KYNA levels. Lines, boxes, and whiskers represent the median, the interquartile range, and the 10th–90th percentiles, respectively. *$p < 0.05$, * significantly different from the naive, † from the VEH, ‡ from the TH group.
